# The Effects of Dorsal Cortical Comminution on Radiographic Results following Percutaneous Pinning for Extra-Articular Colles' Fracture

**DOI:** 10.1155/2015/714351

**Published:** 2015-07-29

**Authors:** Po-Yao Chuang, Tien-Yu Yang, Shih-Hsun Shen, Yao-Hung Tsai, Kuo-Chin Huang

**Affiliations:** ^1^Department of Orthopaedic Surgery, Chang Gung Memorial Hospital, Chiayi 613, Taiwan; ^2^College of Medicine, Chang Gung University, Taoyuan 333, Taiwan

## Abstract

A number of studies have demonstrated that dorsal cortical comminution (DCC) can predict redisplacement after nonoperative treatment of Colles' fractures; however, the effects of a DCC defect on radiographic outcomes following percutaneous pinning for dorsally displaced extraarticular Colles' fractures are unclear. We, therefore, performed a retrospective study on 85 patients who sustained such fractures treated with percutaneous pinning within 2006–2009. The main outcome measures included four radiographic parameters, including radial height, radial inclination, radial tilt, and ulnar variance. The radiological results showed that all fractures after percutaneous pinning followed the same time series changes and patterns of fracture collapse regardless of the presence of a DCC defect. The use of the pinning construct is to provide support for static loading but not for dynamic loading. Although the final radiographic outcomes were classified as acceptable in fractures with and without DCC, we recommend that a different approach in the management of displaced Colles' fractures might be necessary in consideration of increasing patient expectations of health care.

## 1. Introduction

There are myriad factors that affect clinical results and patient satisfaction following Colles' fracture [1]. Although the relationship between form and function is not invariable, the goal of surgical treatment for displaced extra-articular Colles' fractures should be to reconstruct and to keep the radial alignment until bone healing [2, 3]. Failed restoration and maintenance of the anatomy results in malunion of the distal radius, which may affect the biomechanics of the radiocarpal joint and the distal radioulnar joint and thus result in pain at the wrist, loss in range of motion, and/or decreased grasp strength [4, 5]. In a retrospective study on Colles' fractures after reduction with and without redisplacement, Chang et al. found that patients with malunion would have a lower rate of satisfactory functional outcome than those without it (68% versus 82%) [6]. A number of radiographic measures are used in the assessment of Colles' fractures, including radial height (RH), radial inclination (RI), radial tilt (RT), and ulnar variance (UV). Based on these four parameters, Graham suggested and spread the evaluation criteria, which has become one of the most widely used guidelines for treatment of patients with displaced Colles' fractures [2].

Percutaneous pinning remains a key method of surgical fixation for Colles' fractures. Although there is some evidence to support its use, the precise role of percutaneous pinning is not established [7, 8]. The reliability of this method to achieve and maintain the reduced position until bone healing is a major concern [9, 10], particularly in patients suffering from dorsally displaced fracture with dorsal cortical comminution (DCC). Lafontaine et al. [11] indicated that Colles' fractures with DCC are highly unstable and tend to suffer redisplacement following closed reduction. Mackenney et al. [12] reported that early/late instability and malunion occurred three to six times more frequently in fractures with DCC compared with those without. Although a number of clinical and biomechanical studies [12–15] have regarded metaphyseal comminution as a potential predictor of fracture instability, little is known about the effect of DCC on radiographic outcomes following percutaneous pinning for displaced extra-articular Colles' fractures. This study was thus designed to compare the time series changes and patterns of fracture collapse after percutaneous pinning between fractures with DCC and those without. Our objective was to explore the metaphyseal stability in patients with Colles' fractures after percutaneous pinning.

## 2. Patients and Methods

### 2.1. Participating Patients, Inclusion/Exclusion Criteria, and Study Significance

After being approved by the Institutional Review Board (IRB) of Chang Gung Memorial Hospital (CGMH), this study included all adult patients with displaced extra-articular Colles' fractures (AO/OTA type 23-A2.2 or 23-A3) who were treated with percutaneous pinning in our institution from 2006 to 2009 [16]. The authors excluded patients with bilateral Colles' fractures, open or multiple fractures, intra-articular fractures, palmarly displaced fractures, underlying bone pathologies, and medical problems that could severely jeopardize bone healing and self-care capability. At the time of study enrollment we summarized the collected data, which included demographics, fracture types, surgical methods, and radiographic measurements. These data were analyzed to study the time series changes and patterns of fracture collapse and the causal link between DCC and treatment outcomes. Results of this analysis may contribute to refinement of surgical strategy for displaced Colles' fractures.

### 2.2. Surgical Techniques and Postoperative Hand Therapy Program

Two methods of percutaneous pinning for displaced extra-articular Colles' fractures were regularly adopted in our institution, including the modified Kapandji and Willenegger techniques [15, 16]. All patients after percutaneous pinning received postoperative hand therapy according to a standardized protocol [16]. The wrist was fully immobilized for 8 weeks in a palmar short-arm splint. Active finger motion and forearm axis rotation were encouraged immediately after surgery, but power grip even in the splint was averted. The Kirschner- (K-) wires were removed from all wrists, as outpatients, ordinarily after 6 weeks. For the next 1 month, physiotherapy emphasizing on stretching and active range of motion exercises for the wrist and forearm was performed with the splint removed only for the time of the hand therapy exercises. After this 1-month period, the splint was discarded and power grip was permitted.

### 2.3. Definition of Treatment Outcome

Regular radiographic assessments were carried out after percutaneous pinning; they were performed immediately after surgery, at one, two, three, six, and twelve months, and before and after removal of the K-wires. Four radiographic parameters including RH, RI, RT, and UV were used as the main outcome measures in this investigation. From the PA film of wrist, RH is defined as the distance between two vertical lines to the long axis of the radius, one drawn at the top of the radial styloid process and the other at the ulnar cape of the lunate fossa. RI, assessed from the PA film, is defined as the angle between a line linking the top of the radial styloid process and the ulnar cape of the lunate fossa and a line drawn vertical to the long axis of the radius. RT, assessed from the lateral film, is defined as the angle between a line linking the dorsal and palmar lips of the distal radial articular surface and a line drawn vertical to the long axis of the radius. UV, assessed from the PA film, is defined as the distance between two vertical lines to the long axis of the radius, one from the distal ulnar articular surface and the other from the ulnar cape of the lunate fossa. Generally, the average values of RH, RI, RT, and UV for the population should be 11 mm, 23°, 11°, and 0 mm, respectively [17].

### 2.4. Statistical Analysis

Fisher's exact tests and *χ* ^2^ tests were used to analyze nominal variables. For numerical variables, Wilcoxon rank sum tests were used for between-group comparisons. Significance was set at *P* < 0.05 (two-sided). SPSS 12.0 (SPSS Inc., Chicago, IL, USA) was used for all analyses.

## 3. Results

### 3.1. Analyses of Patient Characteristics and Related Variables

Eighty-five patients with displaced extra-articular Colles' fractures were enrolled in this 4-year-long investigation. The mean patient age was 58.3 years (range from 20 to 80); twenty (23.5%) were male, and 34 (40.0%) of fractures were the dominant wrist. Twenty-five (29.4%) patients were treated with the modified Kapandji method and 60 (70.6%) were treated with the Willenegger method. Based on the AO/OTA classification system, 30 (35.3%) fractures were classified as type 23-A2.2 (group 1: fractures without DCC) and 55 (64.7%) were classified as type 23-A3 (group 2: fractures with DCC). Comparison of groups 1 and 2 revealed no significant differences in age, gender, and treatment methods (all *P* ≥ 0.160). When the involved wrists were compared between the two groups, there was a significant trend toward an increased incidence of nondominant wrist involvement in group 1 patients (76.7% versus 50.9%, *P* = 0.021) (Table 1). There were no fracture nonunion and tendon or neurovascular complications in this cohort.

### 3.2. Analyses of Radiographic Measurements before and after Fracture Reduction

**Figure 1:**
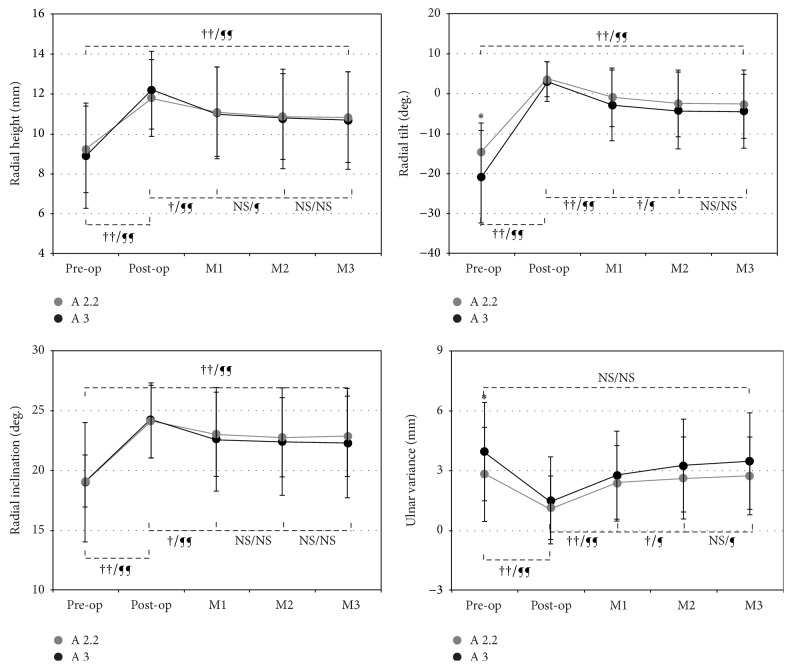
Group comparison of radiographic measurements before and after fracture fixation. Pre-op: preoperatively; post-op: postoperatively; M*x*:  *x* months after the index surgery; ††/¶¶: *P* < 0.001 for A2.2 and A3, respectively; †/¶: *P* < 0.05 for A2.2 and A3, respectively; NS: not significant.

All fractures were diagnosed as displaced Colles' fractures, meaning that the fractured fragments were out of normal palmar tilt in the sagittal plane. The mean (±SD) RH, RI, RT, and UV measures in groups 1 and 2 patients before fracture reduction were 9.22 (±2.19) mm versus 8.90 (±2.66) mm (*P* = 0.551), 19.08 (±4.31) degrees versus 19.01 (±4.99) degrees (*P* = 0.947), −14.67 (±7.46) degrees versus −20.76 (±11.64) degrees (*P* = 0.004), and 2.83 (±2.38) mm versus 3.95 (±2.49) mm (*P* = 0.045), respectively. Intentions to manipulate the fractured fragments back into proper alignment were carried on through the modified Kapandji or Willenegger methods [15, 16]. After fracture reduction and K-wire fixation the mean (±SD) RH, RI, RT, and UV measures in groups 1 and 2 patients were 11.79 (±1.94) mm versus 12.18 (±1.97) mm (*P* = 0.3888), 24.06 (±3.07) degrees versus 24.20 (±3.12) degrees (*P* = 0.847), 3.57 (±4.43) degrees versus 3.12 (±5.09) degrees (*P* = 0.674), and 1.13 (±1.60) mm versus 1.50 (±2.19) mm (*P* = 0.377), respectively (Table 2). Briefly, group 2 patients had significantly more loss of preoperative alignment in RT (−14.67 ± 7.46 degrees versus −20.76 ± 11.64 degrees, *P* = 0.004) and UV (2.83 ± 2.38 mm versus 3.95 ± 2.49 mm, *P* = 0.045) than group 1 patients. The index surgical methods could effectively help restore the acceptable radial geometry in patients suffering from displaced extra-articular Colles' fractures with and without DCC (Table 2 and Figure 1).

### 3.3. Analyses of Radiographic Measurements after Fracture Fixation

**Figure 2:**
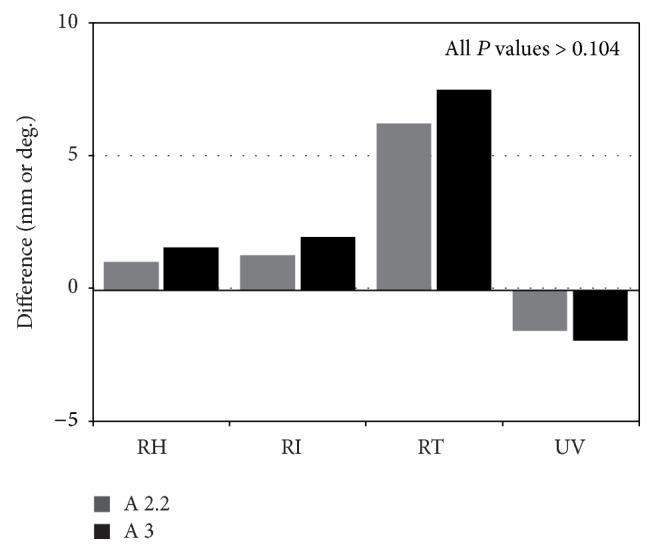
Group comparison of the final loss of alignment after fracture fixation. RH: radial height; RI: radial inclination; RT: radial tilt; UV: ulnar variance.

All fractures were clinically and radiographically healed at the time of follow-up examinations three months after surgery. To clarify the time series changes and patterns of fracture collapse after percutaneous pinning in the two groups, we analyzed the radiographic measures on the wrist films at follow-up. Comparison of groups 1 and 2 revealed no significant differences in all four measures at one, two, and three months after surgery (all *P* ≥ 0.127) (Table 3). We further assessed the monthly changes of radiographic measures and the final loss of alignment after fracture fixation and found that there were no differences between the two groups (all *P* ≥ 0.104) (Table 4 and Figure 2). That is, a similar pattern of fracture collapse occurred in patients after percutaneous pinning for displaced extra-articular Colles' fractures regardless of the presence of DCC. Considering the time series changes of fracture collapse, our results revealed that there was a significant trend in both groups toward recurrent collapse in the first two months after fixation compared to thereafter. Although percutaneous pinning methods significantly improved the radiographic results in RH, RI, and RT (all *P* < 0.001), they did not reconstruct and keep the UV until bone healing (both *P* ≥ 0.096) (Figure 1).

## 4. Discussion

Our study revealed that percutaneous pinning is an effective intervention for the treatment of displaced extra-articular Colles' fractures regardless of the presence of DCC. Actually, the final radiographic results of these two conditions with and without DCC were both described as acceptable without between-group differences in terms of the four radiographic measures (RH, RI, RT, and UV) according to the criteria of Graham [2]. In this cohort, we observed that there was an increased incidence (60% totally) of nondominant wrist involvement in fractures with and without DCC (50.9% versus 76.7%, *P* = 0.021). Ashe et al. [18] reported in their cross-sectional study that the bone response to a nondominant fracture might differ from a dominant fracture. They highlighted the muscle bone interaction and recommended early exercise to lessen the effect of fracture immobilization on bone strength and functional measures. With expectations of improving surgical outcomes steadily increasing, it is necessary to understand the chronological changes of radial geometry after percutaneous pinning, especially in the cases with DCC.

Previous studies [12, 19] have reported that DCC can predict redisplacement after nonoperative treatment of distal radius fractures but whether the pinning construct with a DCC defect can also do so is not clear. Our results showed that the impact of DCC on displaced extra-articular Colles' fractures is significant, due to loss of RT and UV (*P* ≤ 0.045 for both). Through the closed reduction techniques used in percutaneous pinning [16], the fractured fragments could be manipulated back into proper alignment in both conditions with and without DCC. Although promising, it was difficult to achieve the normal palmar RT during percutaneous pinning when compared to other radiographic measures. Bartosh and Saldana [20] observed that when traction is used to reduce the fractures, the palmar radiocarpal ligaments tauten first and then lift the distal fragment before the dorsal radiocarpal ligaments exert any towing, thus restricting the capability of closed reduction techniques to reconstruct the normal palmar RT. Because there were no significant differences in postoperative radiographic measures, we therefore suggested that percutaneous pinning could effectively counteract the negative effects of DCC but not “Bartosh's limit” [20]. To overcome the limit, a different approach in the management of fractures of the distal radius may be necessary [21–23].

Early fracture collapse of the radial geometry was observed within the first two months after percutaneous pinning in both conditions with and without DCC. Pinning constructs with a DCC defect followed the same time series changes and patterns of fracture collapse as those without. Our results suggested that the rigidity of pinning construct for displaced extra-articular Colles' fractures might provide adequate* in vivo* mechanical stability of metaphysis under static loading. However, in response to early rehabilitation of the wrist, the pinning construct might sway dynamically with consequent short-term vibration or oscillatory motion and thus result in some degree of fracture collapse before bone healing. Clinical practice guidelines from the American Academy of Orthopaedic Surgeons (AAOS) moderately recommend stable surgical fixation followed by early wrist motion to treat patients with displaced Colles' fractures [24–26]. We, therefore, recommend that refinement of surgical technology/techniques and postoperative hand therapy program may be necessary in future clinical practices and studies.

There are several limitations in this study. First, it is retrospective. Second, there is lack of functional outcome measures in this study. For treatment of displaced extra-articular Colles' fractures, percutaneous pinning methods have been a well-documented surgical technique. The correlations between the functional results and the radiographic outcomes measures are evident. We thus performed this study to determine the effects of DCC on the maintenance of the reduced position until bone healing, leaving functional outcome considerations aside. Lastly, only AO/OTA type 23-A2.2 or 23-A3 fractures were included in this investigation. We excluded all intra-articular fractures of the distal radius in the current work in order to reduce systemic errors and bias to the smallest possible degree.

## 5. Conclusion

For displaced extra-articular Colles' fractures treated with percutaneous pinning, our time series study revealed that all fractures followed the same time series changes and patterns of fracture collapse regardless of the presence of a DCC defect. The pinning construct could counteract the negative effect of DCC on maintenance of radial geometry; however, it could not overcome the dynamic loading associated with early wrist motion. In consideration of increasing patient expectations in health care, we recommend that refinement of surgical technology/techniques and postoperative hand therapy program may be necessary in future clinical practices and studies.

## Figures and Tables

**Table 1:** Group comparisons of patient characteristics.

Variables	Group 1 (*n* = 30)	Group 2 (*n* = 55)	*P* value^†^
Age, mean years (range)	58.6 (20, 73)	58.2 (23, 80)	0.889
Gender			0.975^‡^
Male	7 (23.3)	13 (23.6)	
Female	23 (76.7)	42 (76.4)	
Side			0.021^‡,*∗*^
Dominant	7 (23.3)	27 (49.1)	
Nondominant	23 (76.7)	28 (50.9)	
Technique			0.160^‡^
Willenegger	24 (80.0)	36 (65.5)	
Kapandji	6 (20.0)	19 (34.5)	

^†^Wilcoxon rank sum test, unless otherwise stated.

^‡^Fisher's exact or chi-squared tests as applicable.

^*∗*^The difference is significant (*P* < 0.05).

**Table 2:** Group comparison of radiographic parameters before/after fracture reduction.

Variables	Group 1 (*n* = 30)	Group 2 (*n* = 55)	*P* value^†^
Pre-op (mean, SD)			
RH^a^ (mm)	9.22 (2.19)	8.90 (2.66)	0.551
RI^b^ (degree)	19.08 (4.31)	19.01 (4.99)	0.947
RT^c^ (degree)	−14.67 (7.46)	−20.76 (11.64)	0.004^*∗*^
UV^d^ (mm)	2.83 (2.38)	3.95 (2.49)	0.045^*∗*^
Post-op (mean, SD)			
RH (mm)	11.79 (1.94)	12.18 (1.97)	0.388
RI (degree)	24.06 (3.07)	24.20 (3.12)	0.847
RT (degree)	3.57 (4.43)	3.12 (5.09)	0.674
UV (mm)	1.13 (1.60)	1.50 (2.19)	0.377
Δ^e^ Post−Pre-op (mean, SD)			
RH (mm)	2.57 (1.70)	3.28 (2.05)	0.093
RI (degree)	4.98 (3.26)	5.19 (3.87)	0.796
RT (degree)	18.23 (9.19)	23.88 (11.07)	0.014^*∗*^
UV (mm)	−1.70 (1.77)	−2.46 (2.08)	0.083

^†^Wilcoxon rank sum test, unless otherwise stated.

^*∗*^The difference is significant (*P* < 0.05).

^a^RH: radial height; ^b^RI: radial inclination; ^c^RT: radial tilt; ^d^UV: ulnar variance; ^e^Δ  *Y*_2_ − *Y*_1_: change of *Y*.

**Table 3:** Group comparison of radiographic parameters after fracture fixation.

Variables	Group 1 (*n* = 30)	Group 2 (*n* = 55)	*P* value^†^
RH^a^ (mm) (mean, SD)			
Post-op	11.79 (1.94)	12.18 (1.97)	0.388
M1^§^	11.08 (2.25)	11.04 (2.32)	0.937
M2	10.87 (2.16)	10.74 (2.51)	0.813
M3	10.82 (2.29)	10.67 (2.45)	0.778
RI^b^ (degree) (mean, SD)			
Post-op	24.06 (3.07)	24.20 (3.12)	0.847
M1	23.00 (3.55)	22.59 (4.37)	0.640
M2	22.72 (3.37)	22.40 (4.52)	0.708
M3	22.83 (3.37)	22.28 (4.60)	0.531
VT^c^ (degree) (mean, SD)			
Post-op	3.57 (4.43)	3.12 (5.09)	0.674
M1	−0.87 (7.40)	−2.87 (8.93)	0.275
M2	−2.44 (8.47)	−4.20 (9.70)	0.387
M3	−2.65 (8.57)	−4.36 (9.32)	0.397
UV^d^ (mm) (mean, SD)			
Post-op	1.13 (1.60)	1.50 (2.19)	0.377
M1	2.36 (1.92)	2.75 (2.22)	0.393
M2	2.62 (2.08)	3.24 (2.34)	0.217
M3	2.73 (1.97)	3.48 (2.43)	0.127

^†^Wilcoxon rank sum test, unless otherwise stated.

^a^RH: radial height; ^b^RI: radial inclination; ^c^RT: radial tilt; ^d^UV: ulnar variance.

^§^M*x*, *x* months after the index surgery.

**Table 4:** Group comparison of time-dependent change of radiographic parameters after fracture fixation.

Variables	Group 1 (*n* = 30)	Group 2 (*n* = 55)	*P* value^†^
RH^a^ (mm) (mean, SD)			
Δ^e^ Post-op − M1^§^	0.71 (1.28)	1.14 (1.56)	0.179
Δ M1 − M2	0.22 (0.54)	0.30 (0.79)	0.617
Δ M2 − M3	0.05 (0.54)	0.07 (0.45)	0.810
Δ Post-op − M3	0.97 (1.26)	1.51 (1.71)	0.104
RI^b^ (degree) (mean, SD)			
Δ Post-op − M1	1.06 (2.41)	1.61 (2.94)	0.359
Δ M1 − M2	0.28 (1.01)	0.19 (1.20)	0.728
Δ M2 − M3	−0.11 (0.88)	0.13 (0.81)	0.253
Δ Post-op − M3	1.23 (2.50)	1.92 (3.26)	0.282
VT^c^ (degree) (mean, SD)			
Δ Post-op − M1	4.44 (5.10)	5.98 (6.88)	0.285
Δ M1 − M2	1.57 (2.26)	1.34 (2.54)	0.671
Δ M2 − M3	0.21 (1.24)	0.16 (1.28)	0.861
Δ Post-op − M3	6.21 (6.17)	7.48 (7.61)	0.409
UV^d^ (mm) (mean, SD)			
Δ Post-op − M1	−1.23 (1.27)	−1.26 (1.23)	0.927
Δ M1 − M2	−0.27 (0.53)	−0.49 (0.75)	0.161
Δ M2 − M3	−0.11 (0.41)	−0.24 (0.65)	0.239
Δ Post-op − M3	−1.60 (1.28)	−1.99 (1.60)	0.236

^†^Wilcoxon rank sum test, unless otherwise stated.

^a^RH: radial height; ^b^RI: radial inclination; ^c^RT: radial tilt; ^d^UV: ulnar variance; ^e^Δ  *Y*_2_ − *Y*_1_: change of *Y*.

^§^M*x*, *x* months after the index surgery.
